# The Contribution of Lysosomotropism to Autophagy Perturbation

**DOI:** 10.1371/journal.pone.0082481

**Published:** 2013-11-22

**Authors:** Roshan Ashoor, Rolla Yafawi, Bart Jessen, Shuyan Lu

**Affiliations:** Drug Safety Research and Development, Pfizer Inc., San Diego, California, United States of America; IISER-TVM, India

## Abstract

Autophagy refers to the catabolic process in eukaryotic cells that delivers cytoplasmic material to lysosomes for degradation. This highly conserved process is involved in the clearance of long-lived proteins and damaged organelles. Consequently, autophagy is important in providing nutrients to maintain cellular function under starvation, maintaining cellular homeostasis, and promoting cell survival under certain conditions. Several pathways, including mTOR, have been shown to regulate autophagy. However, the impact of lysosomal function impairment on the autophagy process has not been fully explored. Basic lipophilic compounds can accumulate in lysosomes via pH partitioning leading to perturbation of lysosomal function. Our hypothesis is that these types of compounds can disturb the autophagy process. Eleven drugs previously shown to accumulate in lysosomes were selected and evaluated for their effects on cytotoxicity and autophagy using ATP depletion and LC3 assessment, respectively. All eleven drugs induced increased staining of endogenous LC3 and exogenous GFP-LC3, even at non toxic dose levels. In addition, an increase in the abundance of SQSTM1/p62 by all tested compounds denotes that the increase in LC3 is due to autophagy perturbation rather than enhancement. Furthermore, the gene expression profile resulting from *in vitro* treatment with these drugs revealed the suppression of plentiful long-lived proteins, including structural cytoskeletal and associated proteins, and extracellular matrix proteins. This finding indicates a retardation of protein turnover which further supports the notion of autophagy inhibition. Interestingly, upregulation of genes containing antioxidant response elements, e.g. glutathione S transferase and NAD(P)H dehydrogenase quinone 1 was observed, suggesting activation of Nrf2 transcription factor. These gene expression changes could be related to an increase in SQSTM1/p62 resulting from autophagy deficiency. In summary, our data indicate that lysosomal accumulation due to the basic lipophilic nature of xenobiotics could be a general mechanism contributing to the perturbation of the autophagy process.

## Introduction

Autophagy is an evolutionarily conserved self-eating process by which cytoplasmic components, including macromolecules (e.g. long-lived proteins) and organelles (e.g. mitochondria), are delivered to lysosomes and degraded [1]. As a hallmark morphological feature of this dynamic process, double-membrane-bound autophagosomes go through a maturation process to sequester various substrates and fuse with lysosomes to form autolysosomes. Eventually, lysosomes can be reformed from the hybrid organelles [2]. Many components are involved in the autophagosome formation and autophagy-related genes (ATG) especially play an essential role in this process. 

Mounting data has revealed involvement of autophagy in various physiological processes including nutrient supply for survival and quality control of intracellular proteins and organelles. Mice that lack of autophagic activity due to various ATG knockouts either die *in utero* or within 24 h after birth [3,4,5]. The vital role of autophagy in the maintenance of cellular/tissue homeostasis is supported by various conditional knockout studies. For example, the liver-specific Atg 7 knockout mouse showed various liver lesions, including hepatomegaly and hepatocyte hypertrophy [4], and cardiac Atg 5 deficient mice displayed cardiac hypertrophy and left ventricular dilation [6].

Recent efforts have revealed autophagy deregulation in multiple pathological conditions such as neurodegenerative disorders, metabolic diseases, infectious diseases and cancer [1]. One characteristic of neurodegenerative disease is the presence of intracytoplasmic/extracellular aggregates that are often autophagy substrates. Disruption of this substrate degradation process within autolysosomes is believed to be a principal mechanism for development of the disease. Therefore, enhancing the autophagy process seems to be a sound strategy for these conditions. Preliminary data has demonstrated promising therapeutic effects with upregualtion of autophagy using mouse models [7]. Several researchers have conducted extensive screening to seek positive autophagy regulators and a range of interesting chemical leads were identified [8,9,10]. Another therapeutic area that autophagy modulation can significantly impact is cancer. The role of autophagy in cancer development is complex. Autophagy prevents tumor formation during the initial stages, however once tumors are established, autophagy may paradoxically promote tumor growth by protecting them from metabolic stress [1]. This concept led to clinical trials using the chloroquine derivative, hydroxychloroquine, in combination with various cancer therapies for the treatment of cancer [11]. In addition, more potent autophagy inhibitors, such as Lys05, have been identified and have demonstrated single-agent antitumor activity in mouse models [12]. One challenge for autophagy manipulator screening is to discern enhancers from inhibitors. Both types of agents can similarly increase the abundance of LC3-II, a widely used marker for autophagosomes, either by increasing autophagy formation or by blocking autophagy degradation downstream. LC3 evaluation is typically complemented with additional evidence, including SQSTM1/p62 level assessment or tandem mRFP/mCherry-GFP fluorescence microscopy to estimate the autophagic flux. 

Lysosomes are the final destination where autophagosomes deliver materials for degradation. Lysosomes are membrane-enclosed compartments filled with acid hydrolytic enzymes (e.g. cathepsins) used to digest macromolecules, and are found in the cytosol of nearly all mammalian cells. For the optimal activity of the acid hydrolases, lysosomes require the maintenance of a low internal pH of about 4-5. The acidic pH in the lumen is achieved by the vacuolar H^+^ ATPase, which uses the energy of ATP hydrolysis to pump H^+^ into the lysosome. The pH gradient between lysosomal lumen and the cytosol can drive hyper-accumulation of basic lipophilic compounds via pH partitioning [13]. The compounds that accumulate in lysosomes are classified as lysosomotropic agents.

Previously, we demonstrated that compounds with certain physicochemical properties (basic pKa > 6.5 and clogP >2) tend to be lysosomotropic [14]. In a separate high content screening study we also demonstrated that many pharmaceuticals falling into this basic lipophilic category cause cell loss, DNA fragmentation, and changes to nuclear size, in addition to the lysosomal mass effects [15]. However, the connection between lysosomal accumulation and autophagy modulation has not been fully explored. Chloroquine, a widely used anti-malarial and anti-inflammatory agent, is a classic lysosomotropic compound. This compound has been shown to inhibit autophagy by increasing pH and blocking the fusion of autophagosomes with lysosomes. Therefore, we hypothesize that basic lipophilic compounds can inhibit autophagy by accumulating in lysosomes (lysosomotropism). Eleven basic lipophilic lysosomotropic compounds, including chloroquine, were selected to evaluate their effects on autophagy modulation. Our results indicate that these selected lysosomotropic compounds behave as autophagy inhibitors. We thus propose the novel concept of lysosomotropism as a general mechanism for predicting autophagy inhibition by basic lipophilic compounds. 

## Materials and Methods

### Test Compounds

All compounds including astemizole, chlorpromazine, chloroquine, clomipramine, desipramine, fluoxetine, imipramine, nortriptyline, paroxetine, sertraline, thioridazine, dimethylsulfoxide (DMSO), bafilomycin A (BFA) and rapamycin were purchased from Sigma Aldrich^®^ (St. Louis, MO).

### Cell Culture

H9c2 cells (CRL-1446) cells were purchased from American Type Culture Collection (Manassas, VA). U2OS cells stably expressing GFP-LC3 (U2OS-GFP-LC3 cells) [16] were a generous gift of Dr. Christina Eng (Pfizer Inc, Pearl River). These cells were maintained in high glucose Dulbecco’s Modification of Eagle’s Medium (DMEM, Life Technologies, Carlsbad, CA) supplemented with 10% (v/v) heat inactivated fetal bovine serum, 100 units/ml penicillin/streptomycin, and 2 mM L-glutamine (Life Technologies, Carlsbad, CA) at 37°C in a humidified air atmosphere at 5% CO_2_. In addition, U2OS-GFP-LC3 cells had 500 µg/ml Gentamicin (Life Technologies, Carlsbad, CA) in the culture media. 

### Viability Assay

Cells were seeded in 96-well plates at 6000 cells/well and grown over night. Cells were then treated with compounds at concentrations of 100 µM, 50 µM, 25 µM, 12.5 µM, 6.2 µM, 3.1 µM, 1.6 µM. The final DMSO concentration was 0.5% in vehicle control and compound-treated wells. Cell viability was measured at 24 h post compound exposure using Cell Titer Glo Luminescent Viability Kit (Promega Corporation, Madison, WI) following the manufacturer’s instructions. The bioluminescence was measured using a Tecan Safire^2^ microplate reader (Männedorf, Switzerland). The IC50 of each compound was determined using GraphPad Prism 5 Software following a sigmoidal dose response curve for variable slope.

### LC3 assessment

#### H9C2 cells

Cells were seeded in 96-well plates at 6000 cells/well and grown over night. Cells were then treated with compounds for 24 h in triplicate wells. The Thermo Scientific LC3 Detection Kit (ThermoFisher, Waltham, MA) was employed to stain native LC3 based on the manufacturer’s protocol. Briefly, cells were fixed with 4% paraformaldehyde for 15 min. After permeabilization and blocking, primary antibody was added and incubated for 1 h at room temperature. After washing in PBS, cells were incubated with secondary antibody (Dylight 488 goat anti-rabbit) and Hoechst for nucleus counter staining. 

#### U2OS-GFP-LC3 cells

Cells were seeded in 96-well plates at 6000 cells/well and cultured over night. Cells were then treated with compounds for 24 h in triplicate wells. Post treatment cells were washed once with HBSS and stained with Hoechst dye for nucleus counter staining. 

#### Image capture and quantification

Culture plate images were captured with an Array Scan V^TI^ 600 automated fluorescence imager (ThermoFisher, Waltham, MA). Cells were digitally imaged using a 20X objective in the Hoechst and GFP (XF-93) channels and quantification of fluorescence intensity from 10 fields/well (up to 350 cells) was conducted using spot intensity algorithm. 

### SQSTM1/p62 evaluation

U2OS-GFP-LC3 cells were seeded at 3x10^4^ cells per well (0.6 mL) in 24 well tissue culture treated flat bottom plates (Corning, Corning, NY) and incubated overnight prior to dosing. Three concentrations were selected for each compound and each concentration was tested in triplicate wells. Concentrations of 15 µM, 30 µM and 50 µM were selected for chloroquine, desipramine, and imipramine. Concentrations of 10 µM, 15 µM and 20 µM were selected for clomipramine, fluoxetine, nortriptyline, and paroxetine. Concentrations of 5 µM, 10 µM and 25 µM were selected for Astemizole. Concentrations of 2.5 µM, 5 µM and 10 µM were selected for chloropromazine, sertraline and thioridazine. Twenty four hours after compound addition, cells were lysed using freshly prepared complete lysis buffer comprised of the following; RIPA Cell Lysis Buffer 2 (Enzo Life Sciences, Farmingdale, NY), Protease Inhibitor Cocktail, Phenylmethylsulfonyl fluoride (PMSF) at 1 mM concentration, and 20 µg/mL concentration of DNase (Sigma Aldrich, St Louis, MO). Cell lysates were used to evaluate SQSTM1/p62 abundance using SQSTM1/p62 ELISA kits (Enzo Life Sciences® Farmingdale, NY) following the manufacturer’s protocol. 

### Gene Expression Profiling

H9c2 cells were plated in 6 well plates at 30,000 cells/well and cultured over night. The cells were then dosed with the following compounds at the corresponding concentrations: chloroquine 50 µM, desipramine 12.5 µM, fluoxetine 12.5 µM, astemizole 3.1 µM, aripiprazole 25 µM, or 0.5% DMSO as a negative control. The samples were duplicated for each concentration. The dosed cells were allowed to incubate for 24 h. Total RNA was extracted using Qiagen RNeasy Mini Kit (Qiagen, Valencia, CA). RNA quality and quantity were assessed with an Agilent 2100 bioanalyzer with an RNA 6000 Nano Chip (Agilent Technologies, Santa Clara, CA). Two hundred ng of RNA sample was used as input into the Affymetrix procedure as recommended by the protocol (http://www.affymetrix.com). The RNA sample was processed with Affymetrix 3’IVT Express kit following the manufacturer’s instructions (Affymetrix Inc., Santa Clara, CA, USA). The fragmented RNA was hybridized to Affymetrix GeneChip Rat Genome 230 2.0 Array (Affymetrix Inc., Santa Clara, CA. USA) following the protocol from Affymetrix GeneChip and scanned by Agilent Gene Chip Scanner 3000 (Agilent Technologies Inc., Santa Clara, CA). 

### Data Analysis

GeneSpring GX (Agilent Technologies Inc., Santa Clara, CA, USA) software was used to analyze data collected from the gene chips. The statistical parameters were set for a one way Anova comparison of each treatment’s gene expression relative to the DMSO control with a Tukey HsD post hoc test. The threshold cut off was a 1.5 fold difference. A list of probe IDs for the genes affected across the treatments was compiled and input into David Bioinformatics Resources 6.7 Database (The Database for Annotation, Visualization, and Integrated Discovery http://david.abcc.ncifcrf.gov/home.jsp) to perform the biological interpretation of differentially expressed genes for functional annotation clustering. The threshold of EASE Score, a modified Fisher Exact P-Value, was generated for gene-enrichment analysis.

## Results

### Compound selection

The purpose of the study is to determine whether compound accumulation in lysosomes could perturb the autophagy process. In our previous study using Lysotracker Red we had identified multiple basic lipophilic compounds that are lysosomotropic [14]. We selected 11 drugs to assess their effect on autophagy with chloroquine serving as a positive control. Most of the drugs selected are for the treatment of central nervous system (CNS) disorders with the exception of astemizole, an antihistamine (Table 1); however they are from different pharmacological classes and carry distinct structure features. Physicochemical properties including clogP (the calculated partition coefficient of the neutral species of the compound between octanol and water) and basic pKa (the logarithm of the dissociation constant of the most basic center of the compound) were analyzed for the drugs. All drugs selected have clogP value greater 4 and basic pKa ranges from 9.0 to 10.5. 

**Table 1:** Physicochemical properties of selected compounds.

**Drug Class**	**Drug**	**SMILES**	**ClogP**	**Basic pKa**
**Antimalarial**	**Chloroquine**	**CCN(CC)CCCC(C)NC1=C2C=CC(Cl)=CC2=NC=C1**	**5.06**	**10.47**
**Antipsychotic**	**Chlorpromazine**	**CN(C)CCCN1C2=CC=CC=C2SC2=C1C=C(Cl)C=C2**	**5.3**	**9.41**
	**Thioridazine**	**CSC1=CC2=C(SC3=CC=CC=C3N2CCC2CCCCN2C)C=C1**	**6**	**9.64**
**Tricyclic Antidepressants**	**Desipramine**	**CNCCCN1C2=CC=CC=C2CCC2=CC=CC=C12**	**4.47**	**10.4**
	**Imipramine**	**CN(C)CCCN1C2=CC=CC=C2CCC2=CC=CC=C12**	**5.04**	**9.49**
	**Nortriptyline**	**CNCCC=C1C2=CC=CC=C2CCC2=CC=CC=C12**	**4.32**	**10**
	**Clomipramine**	**CN(C)CCCN1C2=CC=CC=C2CCC2=C1C=C(Cl)C=C2**	**5.92**	**9.46**
**Serotonin Selective Re-uptake inhibitors (Antidepressants)**	**Sertraline**	**CN[C@H]1CC[C@@H](C2=CC(Cl)=C(Cl)C=C2)C2=CC=CC=C12**	**5.35**	**9.47**
	**Paroxetine**	**FC1=CC=C(C=C1)[C@@H]1CCNC[C@H]1COC1=CC2=C(OCO2)C=C1**	**4.24**	**10.32**
	**Fluoxetine**	**CNCCC(OC1=CC=C(C=C1)C(F)(F)F)C1=CC=CC=C1**	**4.57**	**10.06**
**Antihistamine**	**Astemizole**	**COC1=CC=C(CCN2CCC(CC2)NC2=NC3=CC=CC=C3N2CC2=CC=C(F)C=C2)C=C1**	**5.84**	**9.03**

Note: The clogP and basic pKa values were calculated using ACD/Labs software (www.acdlabs.com).

### Viability reduction

**Figure 1:**
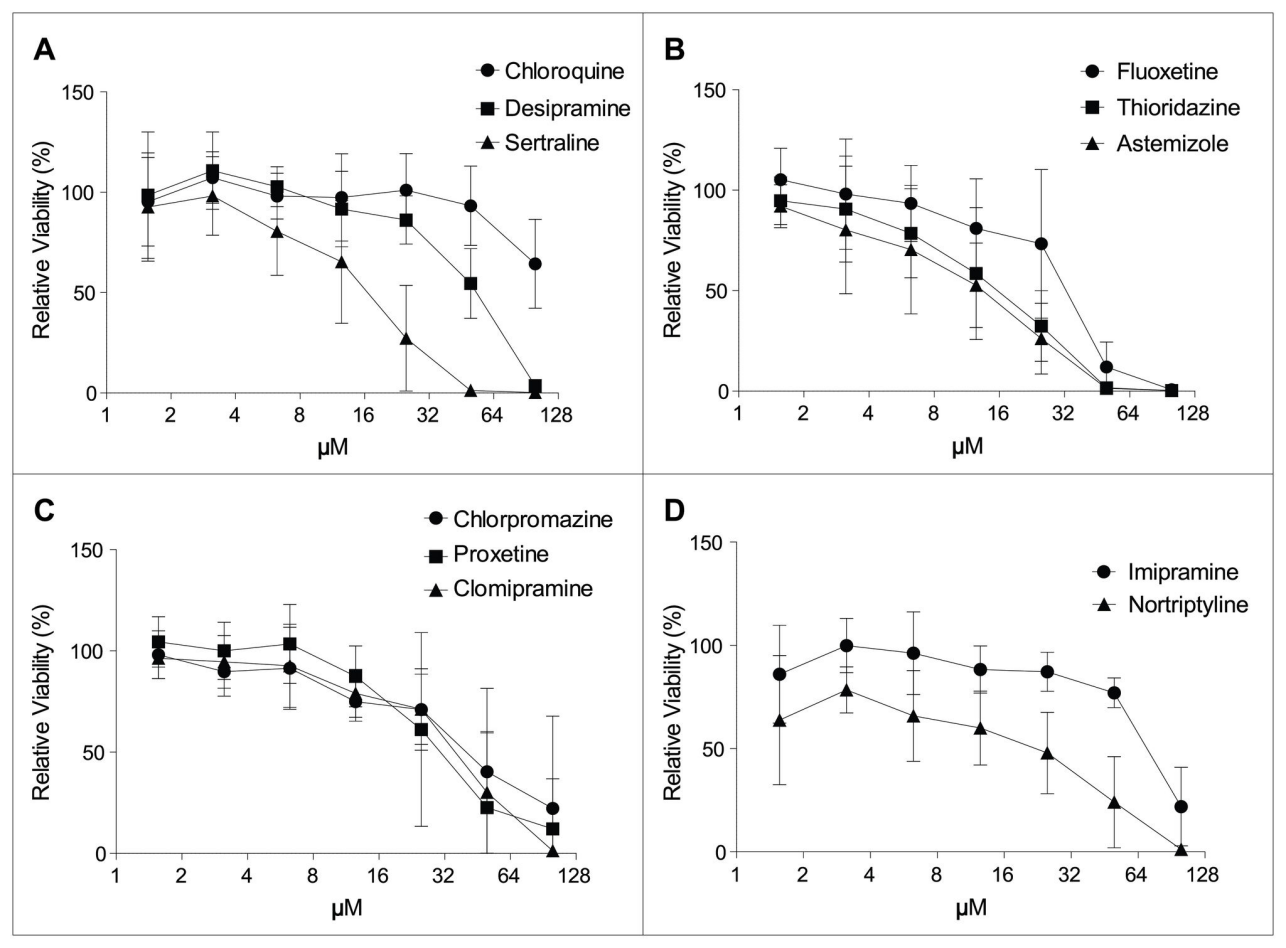
Cytotoxicity induced by lysosomotropic compounds. H9c2 cells were incubated with the test compounds and ATP viability was measured 24 h post compound treatment. Data are expressed as mean ± SD. The mean viability plots were used to calculate IC50s for cytotoxicity (121 µM for chloroquine, 50 µM for desipramine, 15 µM for sertraline, 31 µM for fluoxetine, 14 µM for thioridazine, 11 µM for astemizole, 39 µM for chlorpromazine, 30 µM for paroxetine, 33 µM for clomipramine, 69 µM for imipramine and 13 µM for nortriptyline). The assay was repeated three times.

We first assessed the cytotoxicity of these selected compounds with H9c2 cells using an ATP viability assay. After 24 h of treatment all compounds induced a dose-dependent viability reduction (Figure 1). The IC50 of each compound was determined and chloroquine appears to be the least toxic with an IC50 of >100 µM. Sertraline, thioridazine, and astemizole, along with nortripryline, appear to be among the more toxic compounds with IC50s of 15 µM or less.

### Autophagy evaluation

#### Endogenous LC3

**Figure 2:**
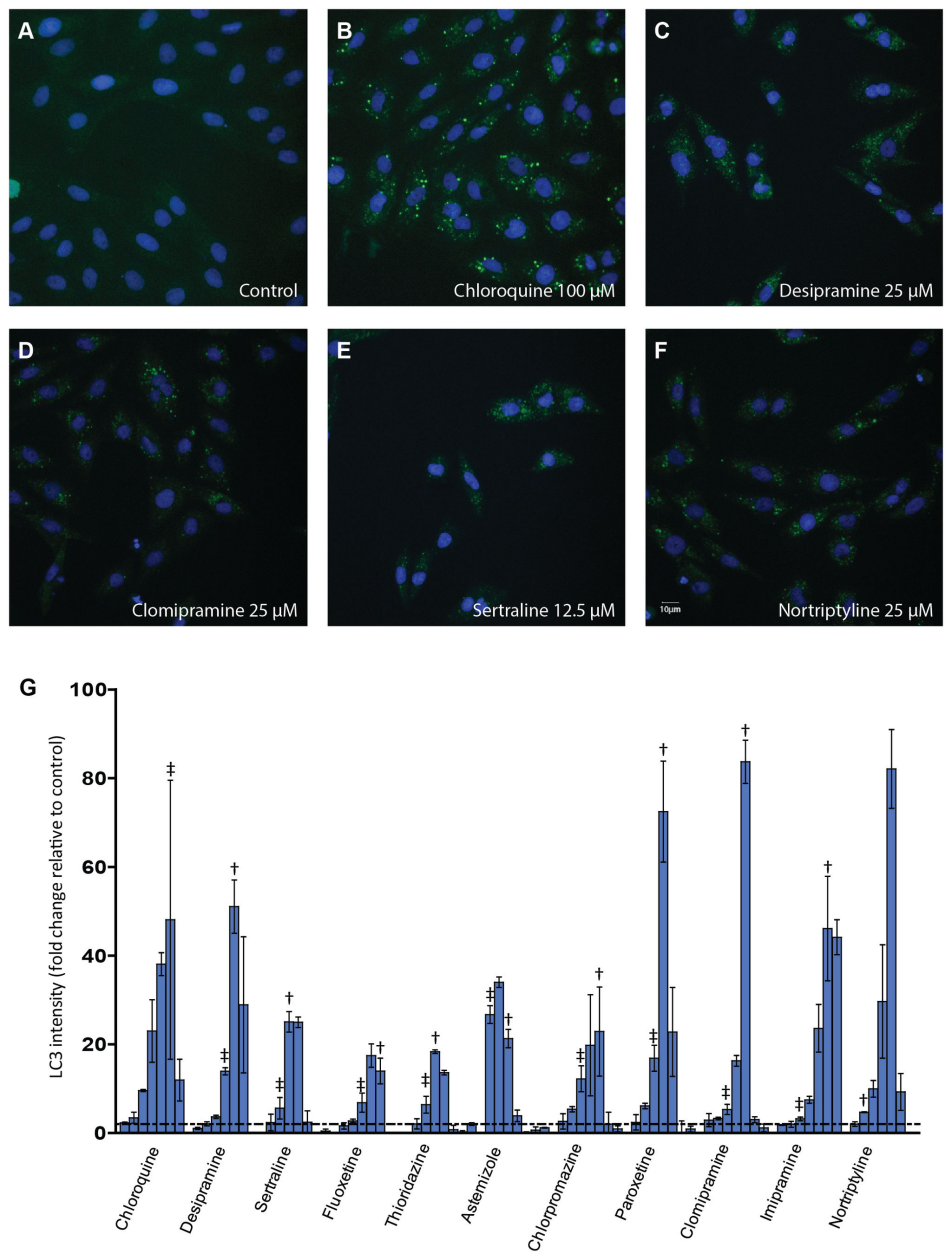
Autophagy modulation by the tested compounds. H9c2 cells were treated with compounds for 24 h. LC3 immunofluorescence staining was conducted to monitor autophagy change. (A-F) Representative LC3 images from the various indicated treatments are shown. (G) Concentration response plots of LC3 change after quantitative image analysis. The fold change of LC3 intensity compared to control was plotted for each compound and data are expressed as mean ± SD. For each compound the concentration rage tested is from 1.5 µM (left) to 100 µM (right). The dotted line depicts 2-fold of the control LC3 intensity. The Assay was repeated three times. †: the maximum concentration with viability > 70%, ‡: the maximum concentration with viability > 90%.

When the autophagosome is formed, the cytosolic Atg 8 protein, also known as LC3, is recruited to the membrane of nascent autophagosomes and controls autophagosome expansion. LC3 is the most widely monitored autophagy–related protein. To evaluate the effects of these selected compounds on autophagy, we first evaluated the expression of endogenous LC3 in H9c2 cells using immunofluorescent staining. All eleven compounds including chloroquine were tested at various concentrations ranging from 100 µM to 1.6 µM. The LC3 staining assay was automated using high-content screening instrumentation programmed to detect and quantify punctate LC3. Visual inspection of images revealed a lack of LC3 puncta in the negative control well and apparent accumulation of LC3 puncta for compound treated wells. Staining images from chloroquine, desipramine, nortripryline, and clomipramine and sertraline treatment are shown as examples (Figure 2). Quantitative LC3 fluorescence intensity of treated wells was employed to calculate the fold change of the LC3 intensity from control wells. All compounds tested exhibited concentration-dependent activity ranging from 17- to 83-fold increased punctate LC3 fluorescence intensity at their optimal concentrations (Figure 2G). The LC3 response followed a biphasic pattern over the concentration range used. When examined closely, it was observed that a ≥ 2-fold increase in LC3 staining occurred at multiple concentrations that demonstrated at least 90% viability, indicating that the LC3 response occurred at non-toxic or minimally toxic concentrations. Interestingly, for quite a few compounds such as desipramine, sertraline and clomipramine, the maximum response occurred at the concentration that had about 70% viability. Generally, reduced response compared to the maximum change of each compound occurred at higher concentrations, which are associated with more severe cytotoxicity (i.e. > 30% loss of cell viability). 

#### GFP-LC3

**Figure 3:**
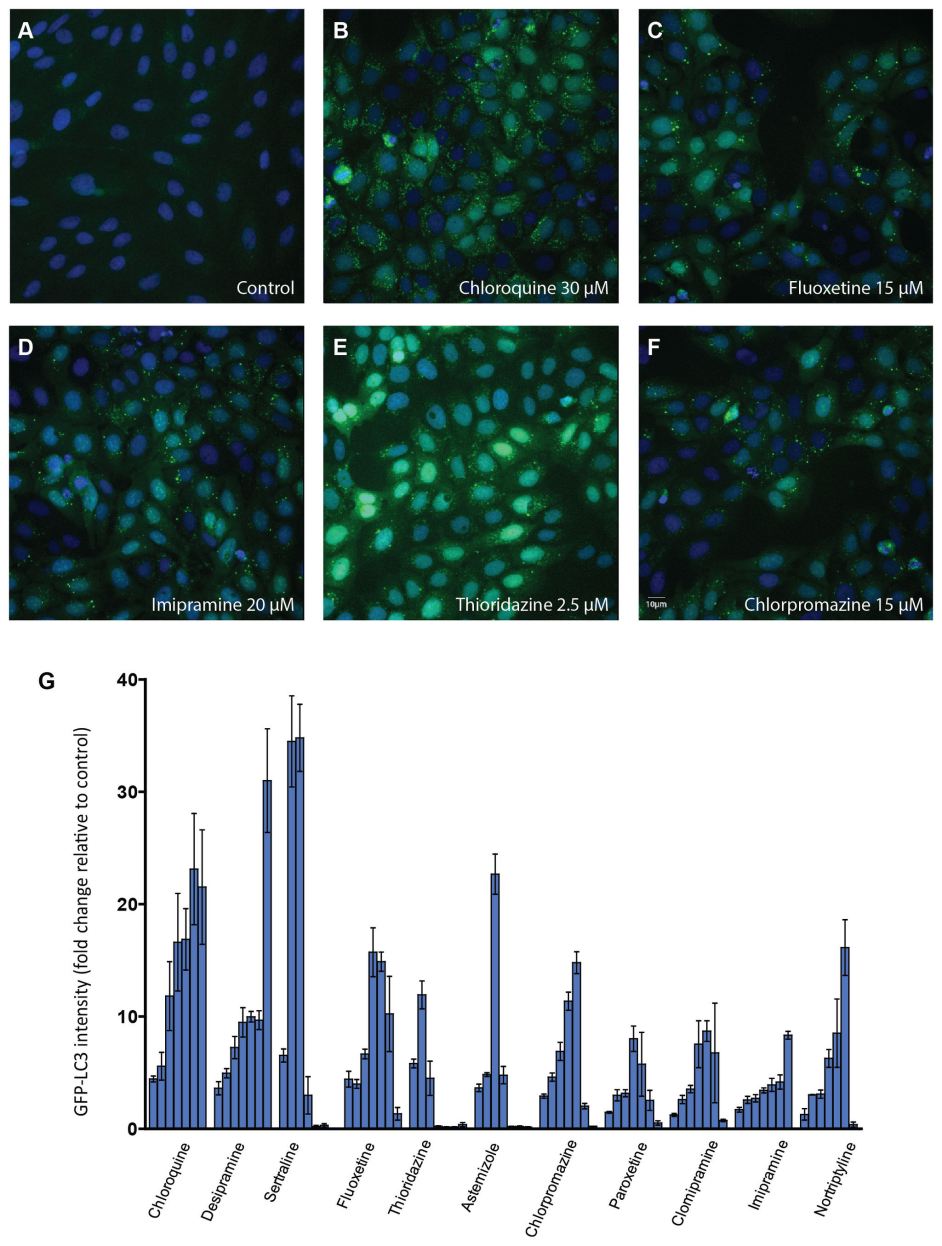
Punctate GFP-LC3 accumulation upon compound treatment. U2OS cells stably expressing GFP-LC3 were incubated with compounds for 24 h. GFP-LC3 and nuclei were visualized by automated microscopy as described in Materials and Methods. (A-F) Representative images from the various indicated treatments are shown (G) Concentration response plots of GFP-LC3 change after quantitative image analysis are shown. The fold change of GFP-LC3 intensity compared to control was plotted for each compound and data are expressed as mean ± SD. For each compound the concentration rage tested is from to 2.5 µM (left) to 50 µM (right). The assay was repeated three times.

Recently, exogenous LC3 tagged with fluorescence has been used to screen autophagy modulators [10]. In order to determine if the compounds could modulate exogenous LC3 puncta in a manner similar to the change observed in H9c2 cells, we further evaluated the LC3 response using U2OS that were stably transfected with a plasmid for expression of LC3 linked at its N-terminus to GFP. In control wells, GFP-LC3 fluorescence was largely diffuse throughout the cytoplasm. However, a remarkable increase in GFP-LC3 punctate fluorescence was observed in the compound treated wells as shown in Figure 3. Similarly, like the response in H9c2 cells, all compounds tested showed concentration-dependent activity ranging from 7- to 34-fold increased punctate GFP-LC3 fluorescence intensity at their optimal concentrations (Figure 3G).

### SQSTM1/p62 assessment

A steady-state increase in the number of endogenous LC3 or GFP-LC3 puncta does not necessarily reflect a stimulation of autophagic activity. Treatment with downstream blockers of the autophagy process can produce very similar results. For instance, bafilomycin A1, a V-ATPase inhibitor that can impede autophagosome fusion with lysosomes, also provoked an increase of LC3 puncta [10]. Chloroquine, used as a positive control in our experiment, also has been recognized as an autophagy inhibitor. In both endogenous and exogenous LC3 assessment, chloroquine considerably increases LC3/GFP-LC3 puncta (Figures 2B, 3B). To determine whether the increase of LC3 puncta by the lysosomotropic compounds is due to upstream enhancement or downstream suppression of autophagy, we evaluated the change of abundance of SQSTM1/p62 with compound treatment. Induction of autophagy leads to decreased SQSTM1/p62 abundance, whereas inhibition of autophagy correlates with increased levels of SQSTM1/p62. 

**Figure 4:**
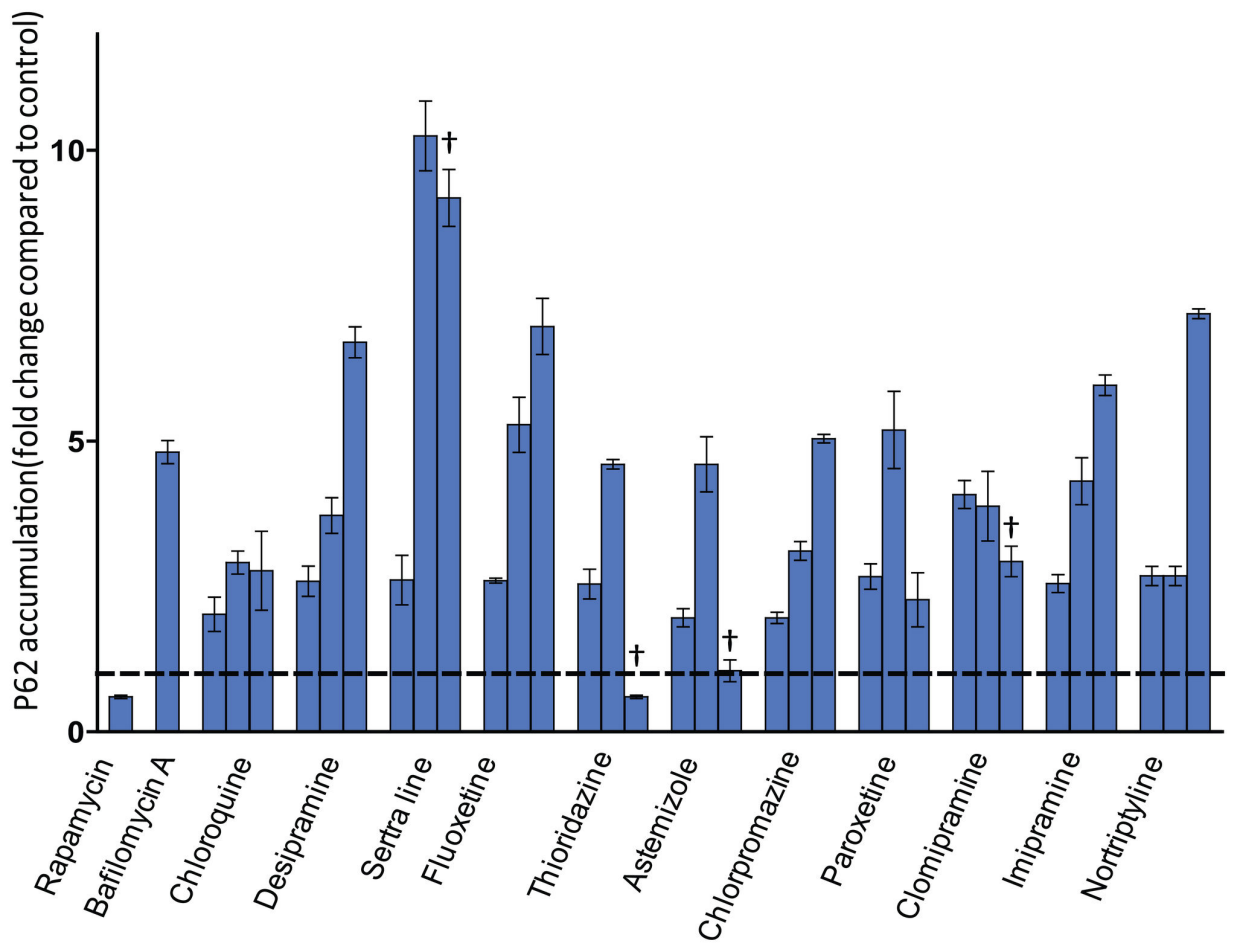
SQSTM1/p62 abundance assessment. U2OS cells stably expressing GFP-LC3 were incubated with compounds for 24 h. SQSTM1/p62 protein level was evaluated using an ELISA assay described in Materials and Methods. The fold change of SQSTM1/p62 abundance compared to DMSO control was plotted for each compound and the data are expressed as mean ± SD. The dotted line depicts the DMSO control level. Rapamycin (1 µM) and bafilomycin A (5 nM) were tested at single concentration. Based on viability profile three concentrations were chosen for the compounds. The concentration range tested is from low (left) to high (right). For chloroquine, desipramine, imipramine concentrations of 15 µM, 30 µM and 50 µM were selected. For clomipramine, fluoxetine, nortriptyline, paroxetine concentrations of 10 µM, 15 µM and 20 µM were selected. For astemizole concentrations of 5 µM, 10 µM and 25 µM were selected. For chlorpromazine, sertraline and thioridazine concentrations of 2.5 µM, 5 µM and 10 µM were selected. All concentrations have viability greater than 60% except the ones labeled with “†”.

U2OS-GFP-LC3 cells were then used to assess SQSTM1/p62 levels. The SQSTM1/p62 ELISA assay was first validated with established modulators of autophagic flux. Stimulation of autophagy by the mammalian target of rapamycin (mTOR) inhibitor rapamycin at a concentration of 1 µM decreased the SQSTM1/p62 level by approximately 40% (Figure 4). In contrast, the autophagy inhibitor, bafilomycin A1, increased SQSTM1/p62 level by approximately 4-fold compared to control cells. Based on the viability profile in U2OS-GFP-LC3 cells (data not shown), three different concentrations, with the relative viability (compared to control) at the highest concentration ≤ 80%, were selected for SQSTM1/p62 assessment. All tested compounds induced a concentration dependent increase of SQSTM1/p62 ranging from 2- to 10-fold at their maximal effect concentrations (Figure 4). The highest concentrations tested for sertraline, thioridazine, astemizole and clomipramine were associated with viability ≤ 60% relative to controls and resulted in lower SQSTM1/p62 abundance compared to their maximal responses. Sertraline seems to be the most potent compound for this particular endpoint. This result suggested that the increased LC3 puncta by the selected lysosomotropic compounds are due to the inhibition of autophagy process. 

### Gene Expression Profiling

To broaden the understanding of the effects of these lysosomotropic compounds on the cells, we selected four compounds to conduct gene array profiling analysis. H9c2 cells were treated with the following: chloroquine (50 µM), desipramine (12.5 µM), fluoxetine (25 µM), astemizole (3.1 µM), or 0.5% DMSO for 24 h. The particular concentration selected for each compound caused between 20%-30% cytotoxicity after 24 h treatment. The various compound treatments produced gene lists ranging from 575 to 1600 probe IDs that had ≥ 1.5 fold changes in expression relative to the DMSO treatment. A list of probe IDs (699) for the genes affected across three or more treatments was compiled and imported into David Bioinformatics Resources 6.7 Database for functional annotation clustering. The output from the David database produced 178 clusters, and the top two clusters with the highest enrichment scores were Annotation Cluster 1 (enrichment score 7.39) and Annotation cluster 2 (enrichment score 5.94) (Table 2). Table 3 contains the annotation terms provided within each cluster along with their corresponding p-values and the overall enrichment score for each cluster. Annotation Cluster 1 contained elements in reference to the extracellular matrix and extracellular region while the terms within Annotation Cluster 2 pertained to structural cytoskeleton and associated proteins. In addition it was noted that the probe IDs/ genes within both clusters were down-regulated across the board. Table 3 provides a summary of sampling of the probe IDs/genes within each cluster. Also included is the corresponding fold change of the gene under each treatment condition. The data indicates a definite down regulation of cytoskeletal, fibrous, and extracellular long-lived proteins.

**Table 2:** Functional clustering of gene expression profiling.

**Annotation Cluster #**	**Terms within cluster**	**Enrichment Score**	**P-value**
**Annotation Cluster 1**		**7.4**	
	extracellular matrix		4.2E-12
	extracellular region part		1.8E-11
	extracellular region		2.5E-10
	proteinaceous extracellular matrix		7.7E-10
	extracellular space		1.1E-04
	extracellular matrix part		2.4E-04
	Secreted		4.6E-04
**Annotation Cluster 2**		**5.9**	
	contractile fiber		4.0E-13
	contractile fiber part		2.0E-12
	myofibril		8.2E-12
	muscle protein		2.4E-11
	sarcomere		2.2E-09
	striated muscle contraction		4.9E-08
	muscle system process		2.5E-07
	muscle contraction		3.9E-07
	actin cytoskeleton		3.3E-06
	I band		9.2E-06
	actin binding		9.8E-06
	cytoskeletal protein binding		9.8E-06
	Z disc		1.4E-04
	cytoskeleton		3.3E-03
	myosin complex		7.9E-03
	cytoskeletal part		4.8E-02
	non-membrane-bounded organelle		1.3E-01
	intracellular non-membrane-bounded organelle		1.3E-01

**Table 3:** Fold change of selected genes from top two annotation clusters.

**Probe ID**	**Gene Title**	**Fold Change**			
		Chloroquine vs. DMSO	Astermizol vs. DMSO	Fluoxetine vs. DMSO	Desipramine vs. DMSO
**Annotation Cluster 1**					
1367749_AT	lumican	-15.7	-3.4	-7.2	-8.3
1380726_AT	Asporin	-8.7	-4.9	-4.5	-4.8
1370956_AT	decorin	-7.8	-2.2	-2.4	-2.4
1372325_AT	elastin microfibril interfacer 1	-6.0	-1.8	-2.4	-3.2
1367700_AT	fibromodulin	-4.8	-4.2	-4.3	-4.6
1375708_AT	Collagen, type XXVII, alpha 1	-3.9	-3.2	-3.1	-4.0
1374779_AT	coagulation factor XIII, A1 polypeptide	-2.8	-2.1	-2.0	-2.4
1368474_AT	vascular cell adhesion molecule 1	-2.8	-1.6	-1.9	-1.9
1381487_AT	angiopoietin 1	-2.2	-1.6	-2.0	-2.3
1368322_AT	superoxide dismutase 3, extracellular	-2.0	-1.9	-2.3	-2.4
**Annotation Cluster 2**					
1388139_AT	myosin, heavy polypeptide 2, skeletal muscle, adult	-6.5	-8.7	-9.6	-8.9
1368415_AT	myosin, heavy chain 3, skeletal muscle, embryonic	-4.0	-2.8	-4.2	-3.8
1387787_AT	myosin light chain, phosphorylatable, fast skeletal muscle	-3.5	-2.0	-2.6	-2.7
1371247_AT	troponin T type 3 (skeletal, fast)	-3.0	-2.1	-2.5	-2.6
1370033_AT	myosin, light polypeptide 1	-3.0	-2.6	-2.6	-3.9
1392976_AT	tropomyosin 2	-2.9	-1.9	-2.6	-2.5
1371293_AT	similar to Myosin light polypeptide 4 (Myosin light chain 1, atrial isoform)	-2.2	-1.9	-2.6	-2.8
1367785_AT	calponin 1, basic, smooth muscle	-2.2	-2.9	-4.0	-5.6
1379463_AT	filamin A interacting protein 1	-1.8	-2.5	-2.8	-2.6
1375303_AT	LIM domain binding 3	-1.8	-2.2	-2.1	-2.6

Upon further visual inspection of the GeneSpring GX fold change data, a few genes controlled by the Nrf2 signaling pathway were noticed to be upregulated. An increase (1.5-2.5 fold) in glutamate-cysteine ligase, aldo keto reductase, thioredoxin reductase, glutathione-S- transferase, and NAD(P)H dehydrogenase quinone1 genes was observed across all treatments (Table 4). 

**Table 4:** Up regulation of Nrf2 related genes.

**Probe ID**	**Gene Title**	**Fold Change**			
		Chloroquine vs. DMSO	Astermizol vs. DMSO	Fluoxetine vs. DMSO	Desipramine vs. DMSO
1370688_at	glutamate-cysteine ligase, catalytic subunit	2.2	1.7	2.0	1.9
1370902_at	aldo-keto reductase family 1, member B8	2.1	2.0	2.3	2.2
1372523_at	glutamate-cysteine ligase, catalytic subunit	1.9	1.7	1.9	2.0
1386958_at	thioredoxin reductase 1	1.7	2.1	2.2	2.4
1367774_at	glutathione S-transferase A3	1.5	2.3	2.6	2.2
1387599_a_at	NAD(P)H dehydrogenase, quinone 1	1.5	1.6	1.8	1.6

## Discussion

Autophagy dysfunction has been shown to play a role in multiple diseases including neurodegeneration, cancer, and metabolic diseases [1]. Due to its therapeutic potential, autophagy modulation attracts a great deal of interest from both academic and pharmaceutical institutions. However, the mechanism and signaling molecules that play a part in autophagy modulation have not been fully deciphered. The aim of this study was to understand if basic lipophilic lysosomotropic compounds could modulate autophagy. To our knowledge, this is the first study to explore how physicochemical properties and lysosomal accumulation contribute to autophagy modulation. Eleven basic lipophilic compounds, including chloroquine as positive control, all demonstrated robust LC3 puncta induction indicating autophagy modulation. In addition, the autophagy response occurred at non-toxic to minimally toxic concentrations precluding the possibility that the response was a result of cytotoxicity. 

An increase of LC3 puncta does not necessarily indicate autophagy enhancement since downstream suppression of autophagy could also instigate a similar effect. The abundance of SQSTM1/p62 was evaluated to determine if the autophagy modulation observed is due to upstream enhancement or downstream blockage. The SQSTM1/p62 is a multifunctional protein characterized initially by its ability to bind atypical protein kinase C. During autophagosome formation, SQSTM1/p62 links between LC3 and polyubiquitinated substrates. Eventually, SQSTM1/p62 and SQSTM1/p62-bound substrates become incorporated into the mature autophagosome and are degraded in autolysosomes. Therefore, the abundance of SQSTM1/p62 protein could be used as a representative marker for the autophagic flux, where decrease of SQSTM1/p62 abundance is associated with autophagy induction and increased level correlates with autophagy inhibition[17] or deficiency[4,6]. The dose-dependent increase in the abundance of SQSTM1/ p62 by all tested compounds therefore supports the hypothesis that the increase of LC3 was due to autophagy inhibition rather than upstream enhancement. The association was further supported by the transcriptional suppression of plentiful long-lived proteins, including structural cytoskeletal and associated proteins, and extracellular matrix proteins. Long-lived proteins are commonly extracellular or cytoskeletal, involved as structural components of tissues, and are believed to be degraded and recycled indiscriminately by autophagy. Measuring the degradation of long-lived proteins has been used to monitor the autophagy flux [17]. When autophagy is inhibited, turnover of long-lived proteins will be hindered, therefore gene expression of those targets are suppressed as a negative feedback response. These gene expression findings are similar to those found during the aging process. Reduction of mRNA expression of matrix molecules, including collagen, in elderly animals was observed in multiple studies [18,19,20,21], and similar down-regulation of cytoskeletal genes, such as actin, was also associated with aging [22,23]. It is noteworthy that the activity of lysosomes is pivotal for aging cells and age-related decline in overall proteolytic activity has been observed in almost all organisms that have been studied [24]. Perturbed autophagy has been considered as a possible molecular mechanisms for aging [25]. The down-regulation of extracellular matrix genes and cytoskeletal genes by both the aging process and the compounds used in the current study is probably the result of ineffective turnover of long-lived proteins. 

Collectively, the data presented here indicate that lysosomotropic compounds behave as autophagy inhibitors. The fact that we observed the endogenous and exogenous LC3 changes in two different cell lines indicates that this mechanism is not cell-type specific. Our data confirm the finding that chloroquine is an autophagy inhibitor, and is consistent with the previous autophagy modulation reported for imipramine [26], fluoxetine [27] and chlorpromazine [28] (although the authors did not specifically investigate autophagy flux in their studies). 

All the selected compounds have been shown to accumulate in lysosomes in our previous study [14], and from the current study we conclude that these lysosomotropic compounds behave as autophagy inhibitors presumably due to lysosomal dysfunction resulting from lysosomal accumulation. The causes of lysosomal dysfunction by lysosomotropic compounds are potentially multiple fold. Lysosomotropic compounds, chloroquine and methylamine, have been shown to increase lysosomal pH drastically (0.5 to 2.0 pH units) after accumulation [29]. In addition, significant pH elevation by chloroquine was observed *in vivo* [30]. This is consistent with our previous study [14] in which lysosomotropic compounds were shown to decrease Lysotracker staining, indicating pH increase in the lysosomes with the compound treatment since Lysotracker requires low pH in order to accumulate in the organelles. An increase in pH would be expected to decrease the lysosomal degradation capability since acidic pH is optimal for lysosome enzyme activity. In addition, an increase of pH can decrease the fusion capability of lysosomes [31]. Indeed, chloroquine has been shown to decrease autophagosome to lysosome fusion [32]. Lysosomotropic compounds have been shown to decrease lysosomal enzyme activity as well. For instance chlorpromazine and chloroquine have been shown to inhibit the lysosomal phopholipases A1 *in vitro* [33,34] and a time- and dose-dependent down regulation of acid ceramidase was also observed for desipramine, chlorpromazine, and chloroquine [35]. Furthermore it was illustrated that multiple lysosomotropic compounds can redistribute the mannose 6-phosphate receptor from the trans-Golgi network to endosomes and concomitantly increase the secretion of lysosomal enzymes, resulting in a decline of intracellular lysosomal enzyme levels [36], which could further exacerbate the lysosomal dysfunction. 

The link between lysosomal dysfunction and autophagy inhibition is robustly supported by the findings from lysosomal storage diseases (LSDs). Lysosomal defects in LSDs are triggered by mutations of soluble lysosomal enzymes, non-enzymatic lysosomal proteins or non-lysosomal proteins that regulate lysosomal functions. Many LSDs are associated with autophagy defects and autophagosome accumulation [37] as a result of lysosomal dysfunction. Additionally, for various pathological conditions, including neurodegeneration, lysosomal and autophagy dysfunction occur concomitantly and both contribute to the disease progression [38,39], further supporting the close connection between lysosomal dysfunction and autophagy inhibition. The autophagy inhibition by these lysosomotropic compounds is possibly a non-specific result of lysosomal dysfunction. Since other degradation pathways, including endocytosis and phagocytosis converge at the level of lysosomes, it is possible that lysosomotropic compounds also impact the endocytosis and phagocytosis process. Interestingly, the lysosomotropic compounds chloroquine and tamoxifen have been shown to decrease phagocytosis activity [40] and chloroquine is recognized as a clathrin-dependent endocytosis inhibitor [41]. Certainly, studies with additional compounds could further strengthen this hypothesis. 

Many late-onset neurodegenerative diseases, including Alzheimer’s and Parkinson’s diseases, are characterized by intracellular protein misfolding and aggregation. Autophagy upregulation is believed to be a powerful strategy to clear the protein aggregation and slow or prevent neurodegeneration. Hence, extensive screening efforts have been conducted to seek for autophagy enhancers. Interestingly, the physical chemical properties of many supposed autophagy enhancers fall into the basic lipophilic region. Amiodarone [10] (basic pKa 8.47, clogP 7.57 [42]), tamoxifen [43] (basic pKa 8.76, clogP 7.1 [42]), amitriptyline [44] (basic pKa 9.4 and clogP 4.92 [42]), verapamil [45] (basic pKa 8.92, clogP 5.2 [42]) and dimebon [46] (basic pKa 9.05, logP 3.4 [47] ) for instance have been presented in the literature as autophagy stimulators. Because of their basic lipophilic properties, these compounds have the ability to accumulate in lysosomes, as has been previously demonstrated for some of these compounds [14]. It was recently shown that lysosomotropic compounds (e.g. chloroquine) induce lysosomal stress and, consequently, provoke transcription factor EB (TFEB) nuclear translocation [48], which could drive expression of autophagy genes and induce autophagy. One common assay format employed is to study the effects of test compound in the presence of protease inhibitors (e.g., E64d) or bafilomycin A using LC3-II as a measure of autophagy flux. In this scenario, greater LC3-II content with combination treatment relative to the test compounds alone is indicative of increased flux through the autophagy process, while a lack of effect of the combo treatment compared to compound alone may suggest a blockade of the process. While lysosomotropic compounds may also directly stimulate autophagy, thereby causing increases in LC3-II in combination relative to compound alone, the autophagy enhancing may also represent adaptive change due to lysosomal dysfunction and without restoring lysosomal function. These types of compounds would therefore be less likely to provide any therapeutic benefit. Dimebon has recently been reported to have failed in clinical trials for Huntington’s and Alzheimer’s diseases [49,50,51]. Although the causes could be complex, lysosomal dysfunction by Dimebon could have potentially contributed to the failure to demonstrate therapeutic benefit, and further evaluation of the autophagy enhancing effects of the drug would be valuable. 

Another noticeable finding is that all the compounds studied are associated with some degree of cell death at concentrations <100 µM and that they all have clogP values >4. This is consistent with our previous finding that the majority of basic compounds with clogP >4 caused cell death at or below this concentration [15]. Although those compounds induced both cell death and modulated the autophagy process, the autophagy perturbation itself may not have been the causative incident. Since the lumen of lysosomes contains large quantities of hydrolytic enzymes, when the lysosomal membrane is damaged the enzymes released could be harmful to the cells. This depends on the degree of membrane permeabilization; necrosis can occur from massive membrane breakdown while apoptosis may result from partial membrane permeabilization [52]. It is well documented that lysosomotropic agents can trigger lysosomal membrane permeabilization [52,53]. We have previously shown that the cell death associated with lysosomotropic compounds can be rescued by Bafilomycin A, which increases lysosomal pH and prevents compound accumulation, further supporting lysosomal accumulation as the cause for cell death [54]. The autophagy modulation and cell death observed in our study might reflect two separate events occurring concurrently with both being triggered by lysosomal accumulation. 

SQSTM1/p62 has been shown to be a multi-domain protein implicated in the activation of various signaling pathways such as NF-κB, apoptosis, and Nrf2 activation [55]. A non-canonical mechanism of Nrf2 activation by autophagy deficiency due to direct interaction between Keap1 and p62 has been recently proposed [56,57]. Essentially, p62 interacts with the Nrf2-binding site on Keap1, a component of Cullin-3-type ubiquitin ligase for Nrf2, and when SQSTM1/p62 accumulates due to autophagy deficiency, it will compete with the interaction between Nrf2 and Keap1, resulting in stabilization of Nrf2 and transcriptional activation of Nrf2 target genes. SQSTM1/p62 abundance increased at the protein level with all the compounds tested in the study. Gene profiling data demonstrated a clear upregulation of multiple Nrf2 regulated genes such as glutamate-cysteine ligase and glutathione-S- transferase, indicating Nrf2 activation that could be triggered by the increase of SQSTM1/p62 due to autophagy inhibition. Our data are consistent with the recent publication that showed arsenic blocked autophagy, increased p62, and activated Nrf2 [58]. In the report it was shown that arsenic dramatically increased the number of GFP-LC3 puncta and prevented autophagy flux. Nrf2 activation by arsenic is diminished when p62 is knocked down; indicating this activation by arsenic is p62-dependent. Interestingly, like lysosomotropic compounds, arsenic has been reported to induce lysosomal membrane permeabilization [59]. Most likely lysosomotropic compounds and arsenic share a similar course of action, which starts with lysosomal dysfunction, then autophagy blockage, ultimately leading to Nrf2 activation mediated by SQSTM1/p62. 

**Figure 5:**
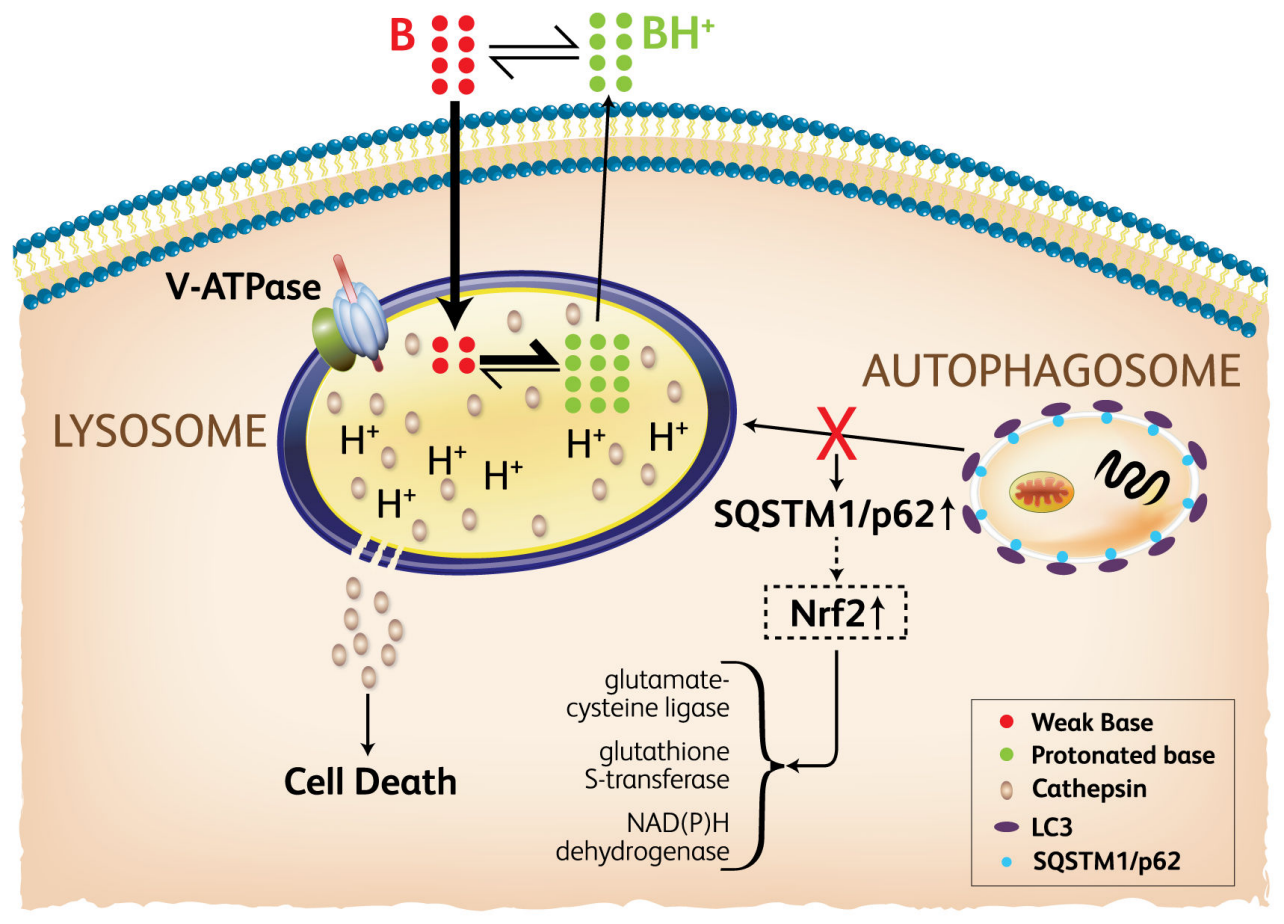
Model of compound accumulation and autophagy perturbation. Basic lipophilic compounds (B) pass through cell membranes and enter lysosomes. The acidic environment within the lysosome causes the majority of the compounds to become protonated (BH) and trapped. Compound accumulation can induce lysosome membrane permeablization and cause cell death. Concurrently, lysosomal dysfunction can potentially occur from a decrease of lysosomal enzyme activity due to either pH changes or direct enzymatic interactions. As a result, the autophagy process could be hindered. An increase of SQSTM1/p62 resulting from autophagy inhibition could further modulate activity of the Nrf2 signaling pathway.

In conclusion, we propose a model (Figure 5) in which basic lipophilic compounds accumulate in lysosomes and not only induce cell death due to lysosome membrane permeablization at higher concentrations, but also perturb autophagy process as a result of lysosomal dysfunction at relatively lower concentrations. The latter activity, we suggest, is likely the result of multiple effects such as decreased lysosomal enzyme activity due to the pH increase or by direct enzymatic inhibition following lysosomal accumulation. Furthermore, upregulation of multiple genes controlled by Nrf2 indicates Nrf2 activation, possibly due to the increase of SQSTM1/p62 by autophagy deficiency. This model has significant implications in autophagy screening and drug induced toxicity. Future investigations, both *in vitro* and *in vivo*, should further explore the dysfunction of other membrane pathways (e.g. endocytosis and phagocytosis) to more fully explore the impact of lysosomotropic agents on cellular processes.
